# Twenty-five years of the *Journal of Thoracic and Cardiovascular Surgery* (2000-2024): Bibliometric insights

**DOI:** 10.1016/j.xjon.2026.101807

**Published:** 2026-05-04

**Authors:** Jiangfeng Yi, Guiqiang Zeng, Litao Li, Lulin Liu, Dechong Zhang, Honglei Wang, Zhenyu Wang, Kuntan Liu, Jinlin Wu

**Affiliations:** a Department of Vascular and Interventional Radiology, Heyuan People's Hospital, Guangdong Provincial People's Hospital, Heyuan Hospital, Heyuan City, Guangdong Province, China; b Department of Cardiac Surgery, Guangdong Cardiovascular Institute, Guangdong Provincial People's Hospital, Guangdong Academy of Medical Sciences, Southern Medical University, Guangzhou, China

**Keywords:** bibliometric analysis, cardiothoracic surgery, *Journal of Thoracic and Cardiovascular Surgery*, citation analysis, research trends

## Abstract

**Objective:**

To analyze research trends and key contributors in *The Journal of Thoracic and Cardiovascular Surgery* over 25 years (2000-2024) using bibliometric methods.

**Methods:**

A total of 9190 articles from Web of Science were analyzed using CiteSpace, VOSviewer, and Bibliometrix to assess publication trends, citation metrics, author/institution/country contributions, co-citation networks, and keyword co-occurrence.

**Results:**

Annual publications increased from 329 (2000) to 659 (2014), then stabilized at an average of 319 ± 32 (2015-2024). Impact factor peaked at 6.44 in 2021. The United States led with 4695 publications, followed by Japan and Canada. Harvard University topped institutional output (662 publications), whereas Blackstone EH was the most influential author (*h*-index: 65). The 20 most-cited articles were predominantly clinical studies focused on surgical techniques and outcomes. Co-citation analysis identified aortic surgery, cardiac valve surgery, and coronary artery surgery as major domains. Citation burst analysis revealed transcatheter aortic valve replacement as the most dynamic recent frontier, with Mack MJ and Popma JJ showing strongest citation bursts. *Cardiac surgery* was the most frequent key word (300 occurrences), followed by *non–small cell lung cancer* and *coronary artery bypass*. Recent emerging terms include *acute kidney injury*, *coronavirus disease*, and *surgical aortic valve replacement*.

**Conclusions:**

This 25-year bibliometric analysis reveals *The Journal of Thoracic and Cardiovascular Surgery* as a leading platform for cardiothoracic surgery research with sustained growth and increasing scholarly impact. The field demonstrates strong North American institutional dominance, with transcatheter aortic valve replacement emerging as the primary research frontier. Future directions should emphasize international collaboration, research in low- and middle-income regions, transcatheter technologies, and translational research integration.

## Graphical Abstract

**Figure:**
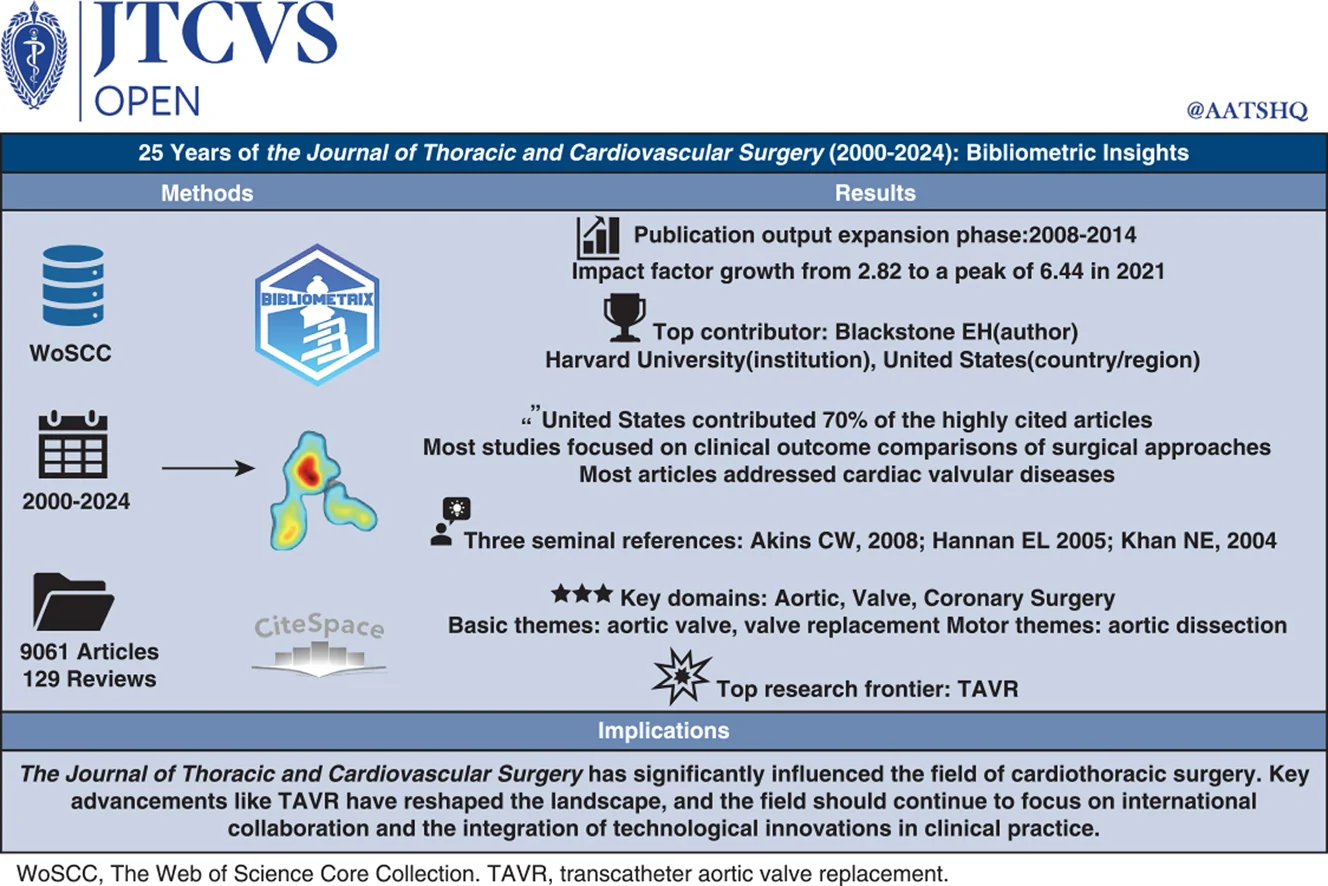

**Figure:**
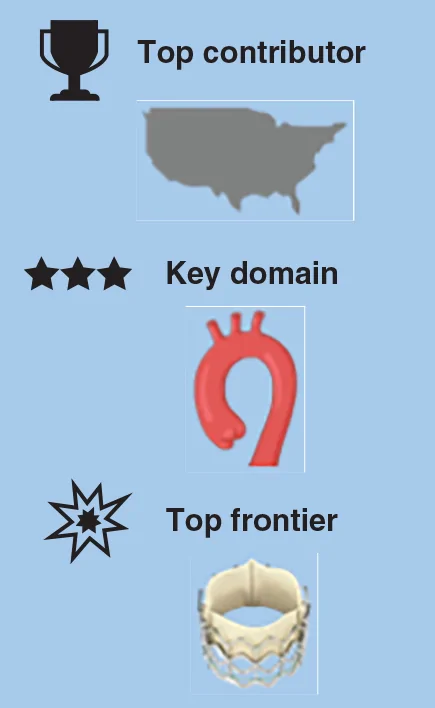
Top contributor in the United States, key domain, and top frontier.

**Central Message**

JTCVS defines the cardiothoracic field, with persistent US dominance and a research landscape increasingly shaped by TAVR.

**Perspective**

Cardiothoracic surgery research evolves rapidly with innovations like TAVR. This comprehensive 25-year bibliometric analysis of JTCVS reveals research evolution trends, key contributors and collaboration network, and future research priorities, helping researchers identify strategic directions and advance the discipline.

*The* *Journal of Thoracic and Cardiovascular Surgery* (JTCVS) is the official publication of both The American Association for Thoracic Surgery and The Western Thoracic Surgical Association. Established in 1931 as *The Journal of Thoracic Surgery*, it became the first English-language publication dedicated to thoracic surgery. The journal was renamed JTCVS in 1959 to reflect advancements in cardiovascular surgery.^1^ JTCVS remains among the most influential journals in the specialty. In the 2024 *Journal Citation Reports*, JTCVS ranked Q1 in 3 categories: 47 out of 230 in Cardiac & Cardiovascular Systems, 19 out of 108 in Respiratory System, and 20 out of 312 in Surgery. Given JTCVS's leading position, a bibliometric analysis will help researchers, editors, reviewers, and clinicians sunderstand the field's key contributors, intellectual foundations, and research frontiers. We systematically analyzed articles published in JTCVS over the past 25 years using 3 bibliometric tools: CiteSpace (Drexel University), VOSviewer (Centre for Science and Technology Studies, Leiden University), and the package *bibliometrix* for R (R Foundation for Statistical Computing).

## Methods

The Web of Science Core Collection (WoSCC) was chosen as the database for this study due to its comprehensive and detailed reference data. On June 8, 2025, we conducted a search and data collection in the WoSCC. The search was limited to the final publication year from January 1, 2000, to December 31, 2024, to cover research spanning the 25-year period from 2000 to 2024. A total of 21,025 documents were retrieved. After excluding document types such as editorial materials, letters, proceedings papers, and corrections, 9190 documents remained, including 9061 articles and 129 review articles. Plain text files containing full records and cited references were exported for subsequent bibliometric analysis. This bibliometric study analyzed publicly available publication metadata from the WoSCC. No human subjects or patient data were involved; therefore, institutional review board approval and patient informed consent were not applicable.

We utilized the Web of Science to extract metrics for countries and institutions, including total publication counts, citation counts, and *h*-indices. The R package *bibliometrix* version 4.3.5^2^ was used to calculate annual publication and author bibliometric indicators, identify the top-20 most-cited JTCVS studies, and conduct a title-based thematic analysis of terminology. We primarily utilized VOSviewer version 1.6.20^3^ to generate collaboration networks for countries, institutions, and authors, including total link strength (an indicator of the collaboration intensity between nodes). We employed CiteSpace version 6.4.R1 Advanced^4^ to generate reference co-citation networks, performing both cluster analysis and citation burst detection to identify intellectual foundations and emerging trends in this field. Due to missing author keywords in 69.8% of Web of Science records, we performed title-based content analysis using CiteSpace. Noun phrases extracted from titles using CiteSpace's Text Processing function served as node types for constructing term co-occurrence networks, followed by clustering and burst detection analyses.

## Results

### Journal Performance

**Figure 1:**
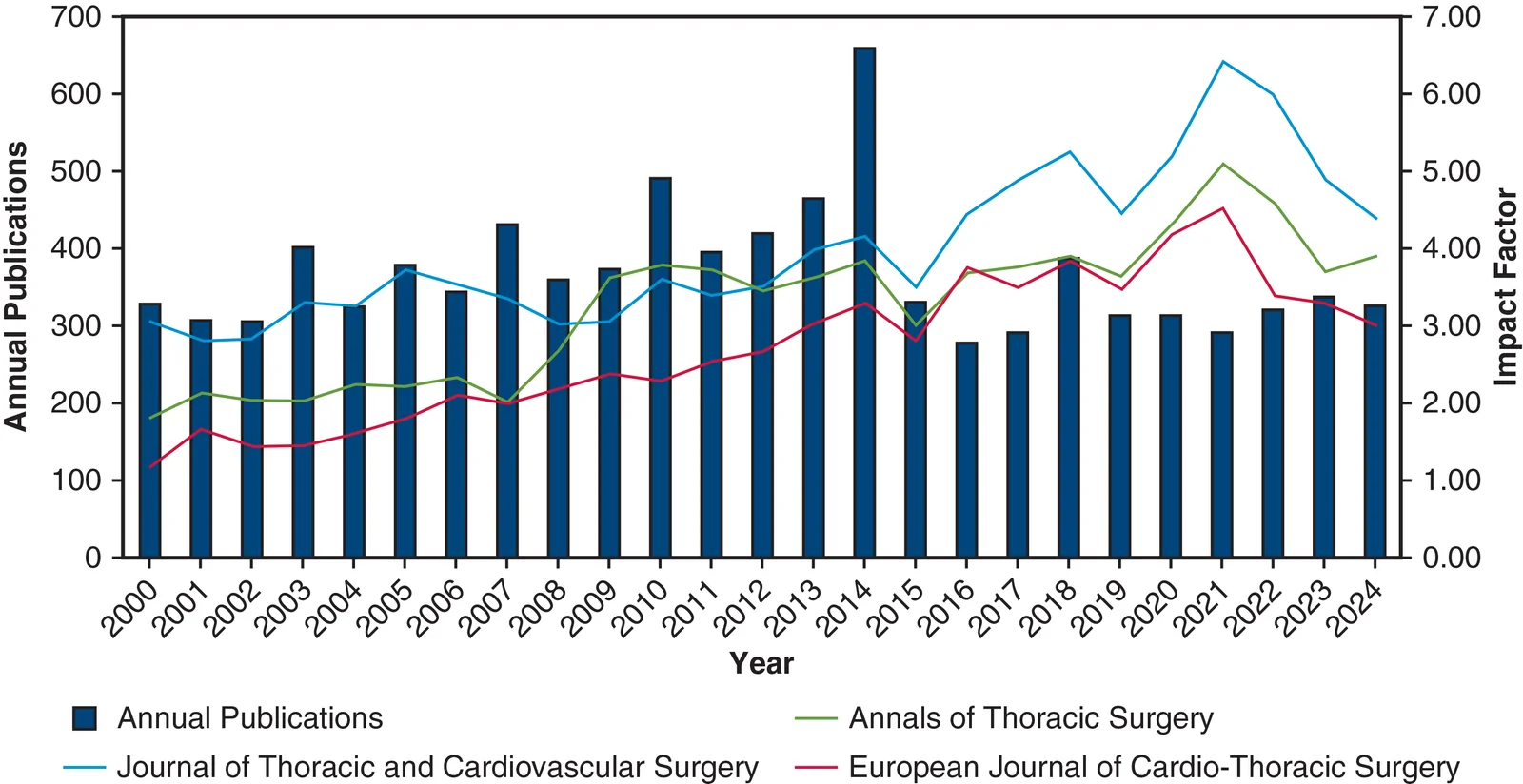
The number of annual publications and journal impact factor from 2000 to 2024.

JTCVS's annual publication output grew steadily from 2000 to 2024, with 3 distinct phases. The early period (2000-2007) showed modest growth with annual publications increasing from 329 to 432 articles. This was followed by a dramatic expansion phase (2008-2014) where publication output surged by 83%, peaking at 659 articles in 2014. The most recent decade (2015-2024) has seen a stabilization phase, with annual publications averaging 319 ± 32 articles. From 2000 to 2024, JTCVS's impact factor showed significant growth from 2.82 to a peak of 6.44 in 2021. Although recent fluctuations resulted in a decrease to 4.40 in 2024, the overall trajectory still demonstrates the journal's growing influence in the field (Figure 1).

### Author Contributions and Collaborative Network

**Figure 2:**
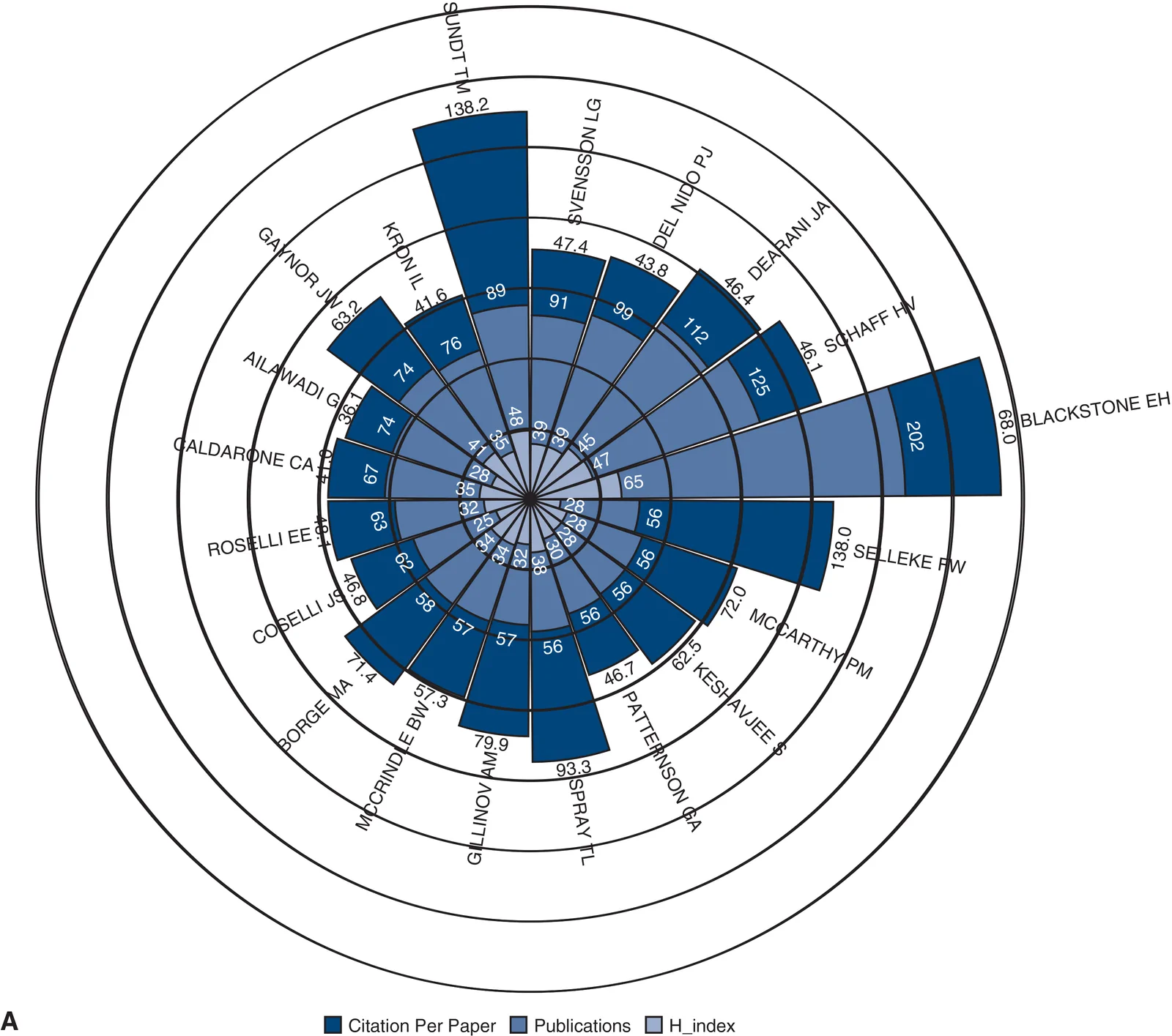

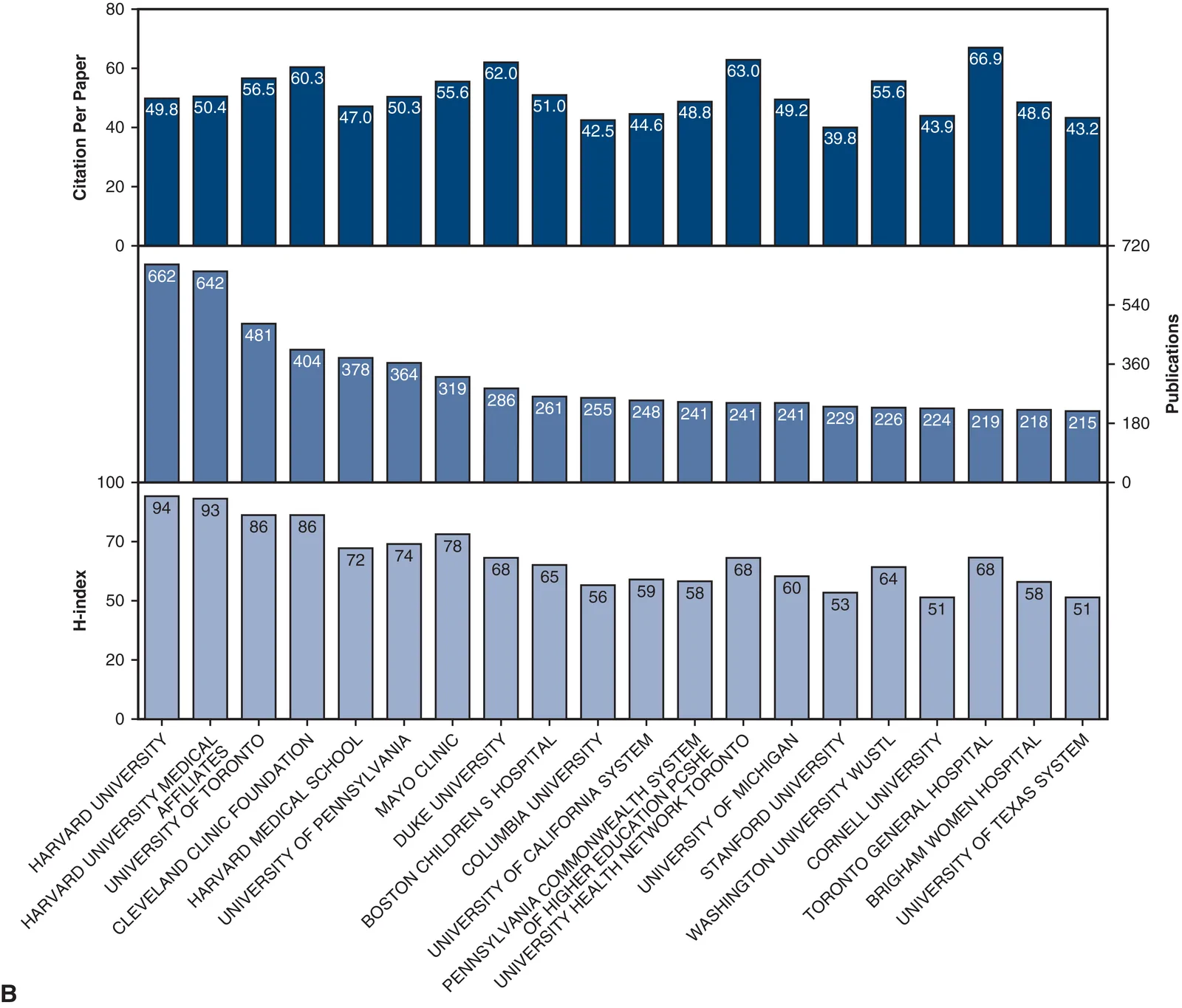
A, The top-20 authors in *Journal of Thoracic and Cardiovascular Surgery* (*JTCVS*). B, The top-20 institutions represented in JTCVS.

**Figure 3:**
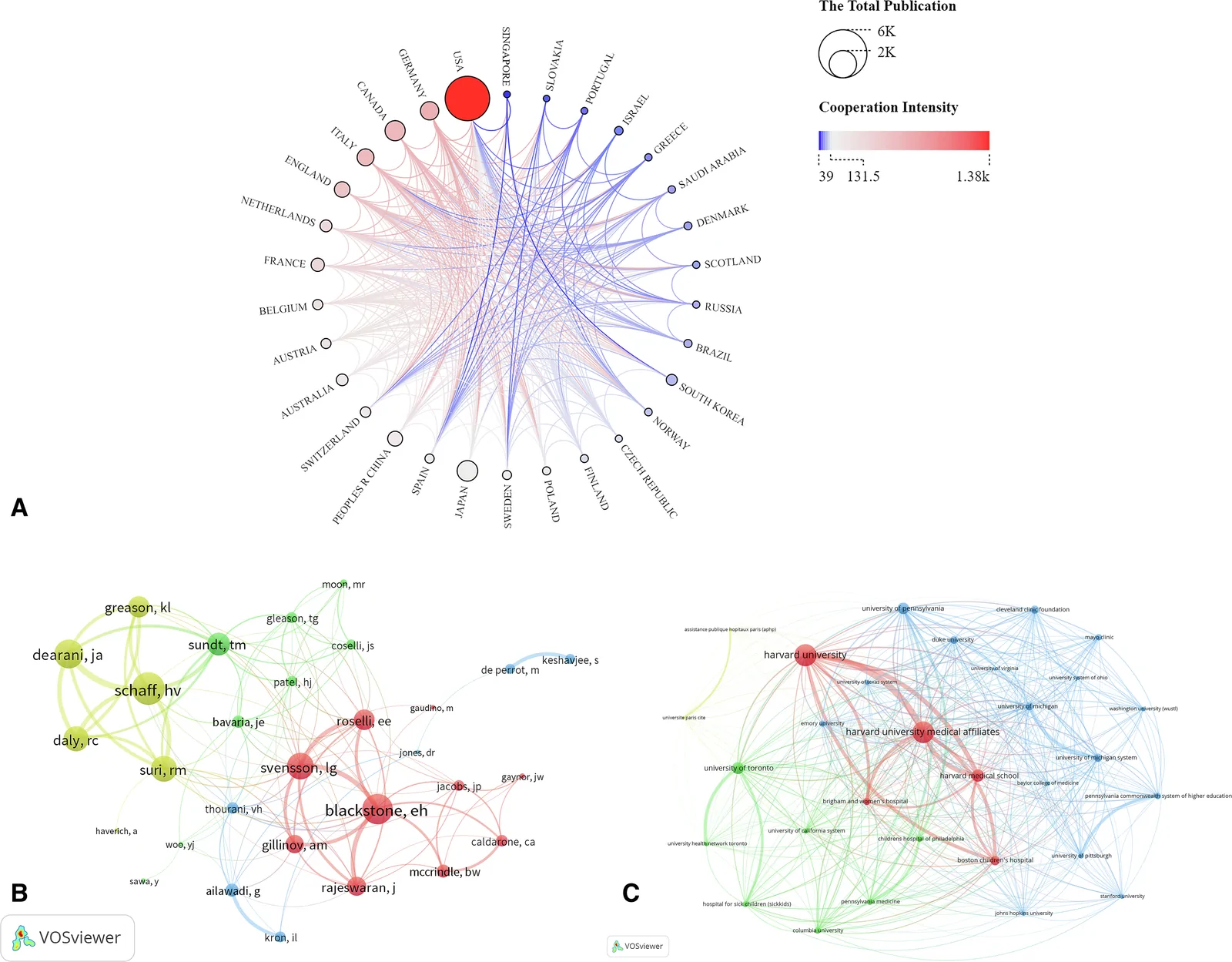
A, The top-30 countries/regions cooperation network in *Journal of Thoracic and Cardiovascular Surgery* (*JTCVS*). B, Authors cooperation network in JTCVS. C, Institutions cooperation network in JTCVS. The collaboration network displays the top-30 authors/institutions with the greatest total link strength. Colors indicate separate collaborative groups. Lines between nodes indicate the frequency of co-occurrence in publications. The size of the node is determined by the total link strength.

From the year 2000 to 2024, a total of 31,264 authors have contributed to the publications in JTCVS. Figure 2, *A*, presents the 20 most influential authors in JTCVS based on multiple citation metrics. Blackstone EH exhibited the highest academic influence, with an *h*-index of 65. Additionally, Blackstone was the most productive researcher, with 202 publications and 68.0 citation per article. Sundt TM achieved the highest citation per article (138.2) with 89 publications and *h*-index of 48. Notably, Sellke FW also demonstrated an exceptional citation per article of 138.0 despite having a relatively modest *h*-index of 22 and 56 publications. The top-3 authors by total citations (Blackstone EH, Sundt TM, and Sellke FW) collectively accounted for 33,773 citations (33.8% of the top-20 authors) from 347 publications (21.9% of the top-20 authors), demonstrating significant concentration of academic influence. Figure 3, *B*, illustrates the author collaboration network. Schaff HV emerges as the most prominent node, exhibiting the highest centrality within the network. He is densely connected with other key figures such as Dearani JA, Suri RM, and Daly RC, forming a tightly knit research cluster with frequent collaborations. Blackstone EH also occupies a central position, serving as a major hub that links to influential authors like Svensson LG and Roselli EE. The network structure reveals distinct groups of researchers who collaborate extensively internally, while also maintaining connections with other subnetworks.

### Institution Contributions and Collaborative Network

Figure 2, *B*, shows the top-20 institutions in JTCVS between 2000 and 2024, revealing significant geographical and institutional concentration. Harvard University exhibited the highest academic influence and productivity, leading with 662 publications, an *h*-index of 94, and citation per article of 49.8. Its affiliated institutions, Harvard University Medical Affiliates (642 publications, *h*-index: 93) and Harvard Medical School (378 publications, *h*-index: 72), ranked second and fifth respectively, further demonstrating the academic dominance of the Harvard medical system. The University of Toronto and Cleveland Clinic Foundation followed, both achieving an *h*-index of 86 with 481 and 404 publications, respectively. Notably, regarding efficiency, Toronto General Hospital achieved the highest citation per article (66.9) despite a lower publication count (219), followed by University Health Network Toronto (63.0) and Duke University (62.0). From a geographical perspective, the United States maintains absolute dominance, securing 17 of the top-20 positions, which reflects a heavy concentration of high-output research centers within the country. Canada serves as the sole non-United States representative, occupying the remaining 3 spots. Notably, despite their smaller representation in terms of volume, Canadian institutions exhibited outstanding research quality through leading citation-per-article metrics. Figure 3, *C*, illustrates the institutional collaboration network. Harvard University appears as the largest node, occupying the central position in the network. It is closely connected with Harvard University Medical Affiliates and Harvard Medical School, indicating a highly integrated research community. Other prominent institutions with significant node sizes include the University of Pennsylvania, the University of Toronto, and Boston Children's Hospital, reflecting their strong collaborative capacities. The dense connections among these top-tier institutions suggest that high-impact research is primarily driven by established academic centers in North America.

### Contributions and Collaborative Networks by Country and Region

A total of 95 countries/regions have published articles in JTCVS. Table 1 presents the top-20 most productive countries/regions in JTCVS research. Total Link Strength measures the intensity of international research collaboration. The United States leads with 4695 publications, an *h*-index of 170, and a Total Link Strength of 1468, demonstrating dominance in productivity, influence, and international collaboration. Japan ranks second in productivity (933 publications, *h*-index 89) but shows relatively lower collaboration intensity (Total Link Strength: 202) and citation impact (38.5 citations per article). In contrast, Canada (third: 891 publications) and Germany (fourth: 719 publications) exhibited higher research quality and connectivity, with *h*-indices of 103 and 96, respectively. Notably, Germany achieved the highest citation per article (51.8), followed closely by Canada (49.8). The network visualization confirms the central position of the United States in global collaborations, with numerous connections to major European research systems. Although Asian contributors like Japan and China show substantial output, they appear more peripherally positioned in the collaboration network compared with the highly interconnected European and North American systems (Figure 3, *A*).

**Table 1:** The top-20 countries/regions in *Journal of Thoracic and Cardiovascular Surgery*

Country/region	*h*-index	Publications	Citation per article	Total link strength
United States	170	4695	44.0	1468
Japan	89	933	38.5	202
Canada	103	891	49.8	733
Germany	96	719	51.8	814
Italy	84	593	46.3	709
ENGLAND	71	494	43.4	638
Peoples Republic of China	54	437	29.3	214
France	64	328	40.3	392
Netherlands	55	248	43.0	425
Australia	50	241	35.3	268
South Korea	42	197	29.9	101
Switzerland	50	171	40.2	220
Belgium	49	155	47.4	333
Austria	45	147	45.9	293
Taiwan	36	131	32.1	35
Spain	39	110	36.4	215
Sweden	41	102	46.5	146
Israel	30	84	31.8	56
Brazil	24	65	32.2	108
Poland	29	61	46.4	131

### Most-often-cited JTCVS Studies

Table 2 lists the top-20 highly cited articles published in JTCVS from 2000 to 2024, with the most prolific period occurring between 2009 and 2011, accounting for 35% of the total. The United States contributed 70% of the highly cited articles, demonstrating its leading position in cardiothoracic surgery research, followed by Germany and Canada with 2 articles each (10%).

**Table 2:** The top-20 most-cited studies in *Journal of Thoracic and Cardiovascular Surgery*

No.	Paper	DOI	First author	Country	Year	Total citations	Total citations per year
1	Consensus-based method for risk adjustment for surgery for congenital heart disease^5^	10.1067/mtc.2002.119064	Jenkins KJ	United States	2002	1066	42.72
2	A classification system for the bicuspid aortic valve from 304 surgical specimens^6^	10.1016/j.jtcvs.2007.01.039	Sievers HH	Germany	2007	850	42.9
3	Continuous insulin infusion reduces mortality in patients with diabetes undergoing coronary artery bypass grafting^7^	10.1067/mtc.2003.181	Furnary AP	United States	2003	772	32.25
4	Isolated aortic valve replacement in North America comprising 108,687 patients in 10 y: Changes in risks, valve types, and outcomes in the Society of Thoracic Surgeons National Database^8^	10.1016/j.jtcvs.2008.08.015	Brown JM	United States	2009	739	41.17
5	Right ventricular failure in patients with the HeartMate II continuous-flow left ventricular assist device: Incidence, risk factors, and effect on outcomes^9^	10.1016/j.jtcvs.2009.11.020	Kormos RL	United States	2010	688	40.47
6	Thoracoscopic lobectomy is associated with lower morbidity than open lobectomy: A propensity-matched analysis from the STS database^10^	10.1016/j.jtcvs.2009.08.026	Paul S	United States	2010	631	37.12
7	Randomized trial of mediastinal lymph node sampling vs complete lymphadenectomy during pulmonary resection in the patient with N0 or N1 (less than hilar) non–small cell carcinoma: Results of the American College of Surgery Oncology Group Z0030 Trial^11^	10.1016/j.jtcvs.2010.11.008	Darling GE	Canada	2011	610	38.13
8	Radical sublobar resection for small-sized non–small cell lung cancer: A multicenter study^12^	10.1016/j.jtcvs.2006.02.063	Okada M	Japan	2006	608	28.95
9	The double-orifice technique in mitral valve repair: A simple solution for complex problems^13^	10.1067/mtc.2001.117277	Alfieri O	Italy	2001	592	22.77
10	An empirically based tool for analyzing mortality associated with congenital heart surgery^14^	10.1016/j.jtcvs.2009.03.071	O'Brien SM	United States	2009	557	31.17
11	Surgical management and outcome of patients with chronic thromboembolic pulmonary hypertension: Results from an international prospective registry^15^	10.1016/j.jtcvs.2010.11.024	Mayer E	Germany	2011	536	33.5
12	Tricuspid valve repair: Durability and risk factors for failure^16^	10.1016/j.jtcvs.2003.11.019	McCarthy PM	United States	2004	526	22.91
13	Neurodevelopmental status at 8 years in children with dextro-transposition of the great arteries: The Boston Circulatory Arrest Trial^17^	10.1016/S0022-5223(03)00711-6	Bellinger DC	United States	2003	511	21.29
14	Comparing apples and oranges^18^	10.1067/mtc.2002.120329	Blackstone EH	United States	2002	503	20.12
15	The American Association for Thoracic Surgery guidelines for lung cancer screening using low-dose computed tomography scans for lung cancer survivors and other high-risk groups^19^	10.1016/j.jtcvs.2012.05.060	Jaklitsch MT	United States	2012	494	32.93
16	Recurrent mitral regurgitation after annuloplasty for functional ischemic mitral regurgitation^20^	10.1016/j.jtcvs.2004.07.037	McGee EC	United States	2004	491	21.39
17	Brain maturation is delayed in infants with complex congenital heart defects^21^	10.1016/j.jtcvs.2008.10.025	Licht DJ	United States	2009	485	26.94
18	Transplantation of hypoxia-preconditioned mesenchymal stem cells improves infarcted heart function via enhanced survival of implanted cells and angiogenesis^22^	10.1016/j.jtcvs.2007.07.071	HuXY	United States	2008	483	25.47
19	Propensity-score matching in the cardiovascular surgery literature from 2004 to 2006: a systematic review and suggestions for improvement^23^	10.1016/j.jtcvs.2007.07.021	Austin PC	Canada	2007	483	24.15
20	Outcomes of 3309 thoracoabdominal aortic aneurysm repairs^24^	10.1016/j.jtcvs.2015.12.050	Coselli JS	United States	2016	477	43.36

Only 1 article was a guideline: the American Association for Thoracic Surgery guidelines for lung cancer screening.^19^ Two methodological studies investigated statistical techniques, including the application of propensity score matching in clinical research to improve causal inference in observational studies.^18^^,^^23^ Only 1 article represented basic experimental research,^22^ whereas clinical studies were the most common type. Among these, 10 studies focused on surgical techniques, primarily compared the effectiveness of different surgical approaches, analyzed surgical outcomes, and evaluated clinical prognosis.^8^^,^^10^, ^11^, ^12^, ^13^, ^14^, ^15^, ^16^^,^^20^^,^^24^

From a research domain perspective, 5 articles addressed cardiac valvular diseases,^6^^,^^8^^,^^13^^,^^16^^,^^20^ 4 focused on lung cancer,^10^, ^11^, ^12^^,^^19^ 4 on congenital heart disease,^5^^,^^14^^,^^17^^,^^21^ and 1 on heart failure and mechanical circulatory support.^9^ One article focused on coronary artery disease,^7^ another described surgical treatment and outcomes in patients with chronic thromboembolic pulmonary hypertension,^15^ and 1 focused on thoracoabdominal aortic aneurysm repairs.^24^

The top-cited article introduced the RACHS-1 risk adjustment method for pediatric congenital heart surgery.^5^ The second most cited article established a classification system for bicuspid aortic valves based on analysis of 304 surgical specimens collected between 1999 and 2003. The system categorized valves into 3 distinct types according to number of raphes, spatial position of cusps or raphes, and functional status of the valve.^6^ The third most cited article demonstrated that continuous insulin infusion reduces mortality in patients with diabetes undergoing coronary artery bypass grafting.^7^

## Reference Co-Citation and Clustering Analysis

### Co-Citation Analysis

**Figure 4:**
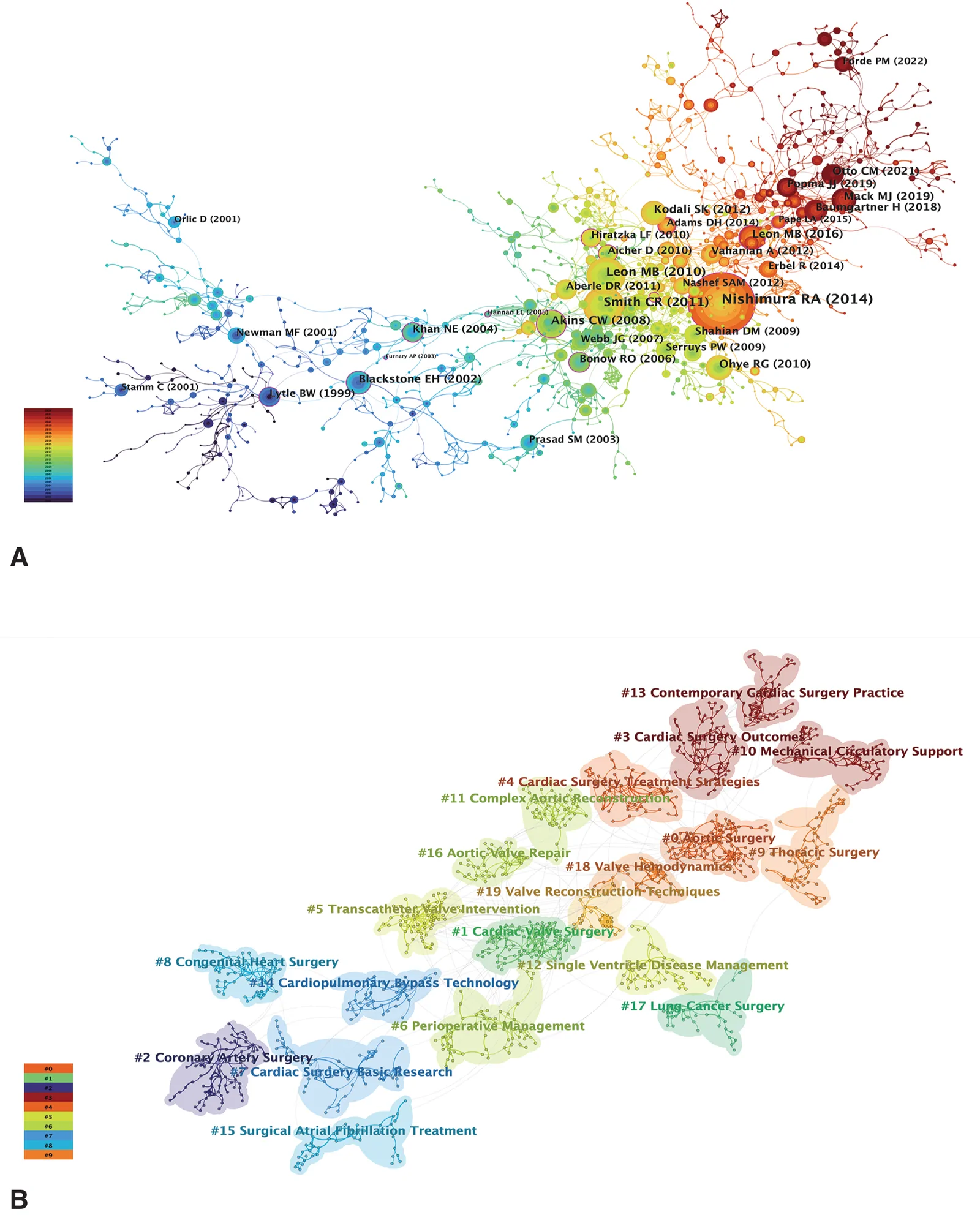

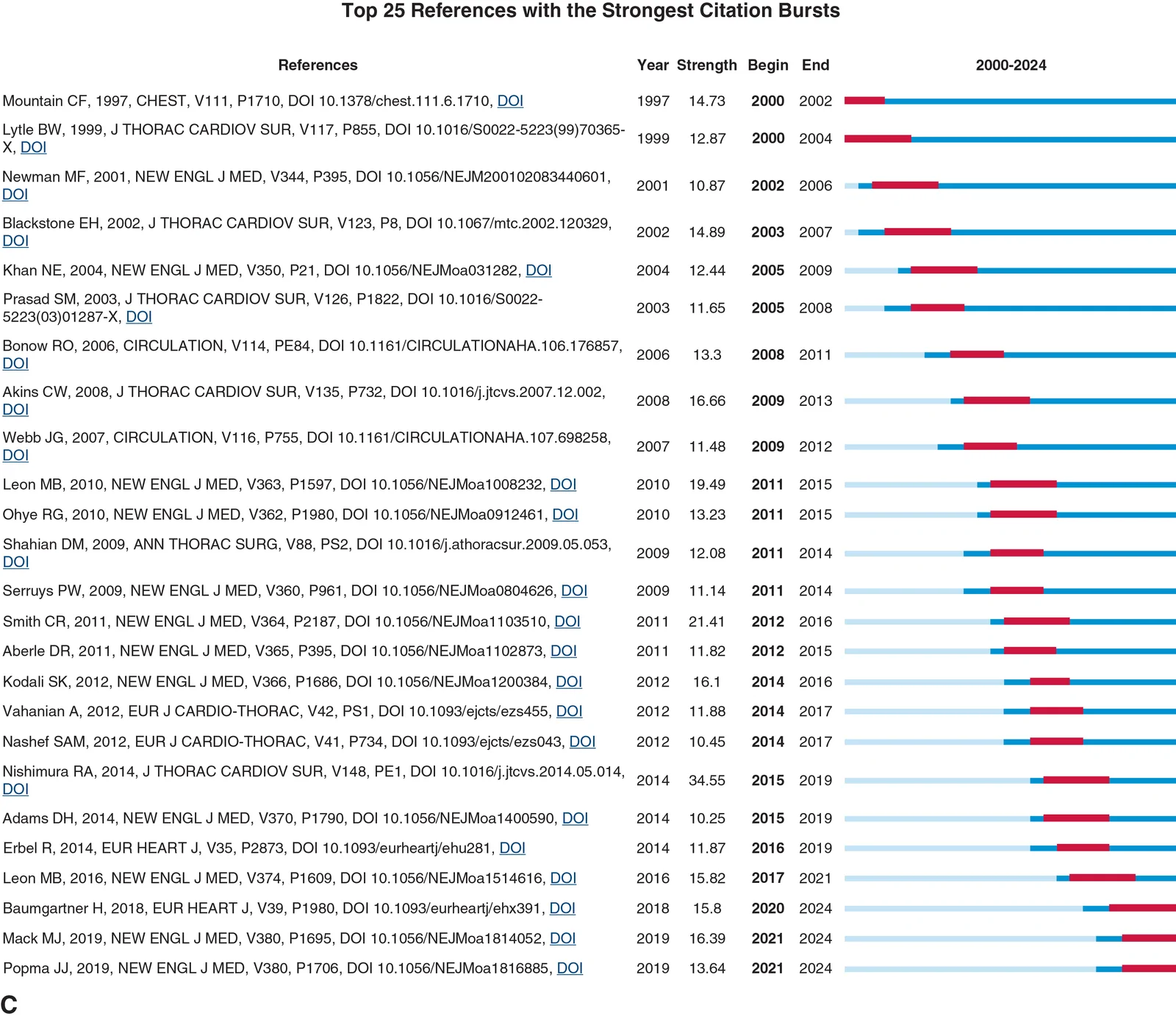
A, Visualization of the co-cited articles in *Journal of Thoracic and Cardiovascular Surgery* (*JTCVS*). B, Clustering visualization of the co-cited articles in JTCVS. C, The top-25 references with the strongest citation bursts. Betweenness centrality indicates critical bridging roles within the co-occurrence network, with higher values signifying greater importance in the network. Purple outer rings indicate values >0.1.

This study conducted a co-citation analysis of literature from the past 25 years to identify key articles that have had significant influence on the research field, revealing the knowledge structure and research frontiers of the domain.^25^ Figure 4, *A*, presents the reference co-citation network with 1661 nodes and 2485 links. Higher betweenness centrality indicates greater influence of the reference. The article with the highest betweenness centrality was Akins CW,^26^ with a value of 0.39, which reported clinical outcomes of various treatment approaches, including medical, surgical, and percutaneous interventional treatment for heart valve disease. Hannan EL^27^ and Khan NE^28^ followed closely with betweenness centrality values of 0.34 and 0.33, respectively.

Table E1 shows the top-10 studies with the highest co-citation rates in this field. The majority of these 10 co-cited articles were published in *The New England Journal of Medicine*, followed by JTCVS. Two were clinical management guidelines for valvular heart disease.^29^^,^^30^ The highest number of articles evaluated transcatheter aortic valve implantation (TAVI), with 5 publications identified, including 2 by Leon MB, indicating this author's outstanding contribution to the TAVI field.^31^^,^^32^ Among these, 4 articles were randomized controlled trials comparing transcatheter aortic-valve replacement (TAVR) with surgical replacement,^32^, ^33^, ^34^, ^35^ and 1 assessed all-cause mortality at 1 year after TAVI in high-risk patients who were not suitable for surgical intervention.^31^ The highly co-cited articles in this field were mainly focused on valvular heart disease topics, particularly TAVI.

### Clustering Analysis

To investigate the primary research domains and developmental trends in JTCVS, we analyzed the reference co-citation network through cluster analysis. This approach groups related publications into distinct clusters representing specific research fields. The cluster size corresponds to the number of publications within each group, with cluster #0 being the largest. Based on the cited literature, JTCVS research is mainly divided into 20 clusters, as shown in Figure 4, *B*.

The largest cluster (#0) was named Aortic Surgery and included 97 members. It mainly discussed: aortic valve surgery, aortic dissection, aortic arch reconstruction surgery, aortic root surgery, brain protection strategies, vascular reconstruction techniques, elephant trunk technique, and thoracoabdominal aortic surgery. Among these, aortic valve surgery primarily focused on TAVR, reflecting recent research hotspots. The most frequently cited article in this cluster was Transcatheter or Surgical Aortic-Valve Replacement in Intermediate-Risk Patients,^32^ cited 31 times. This study provided evidence for expanding the use of TAVR.

The second largest cluster (#1) was named Cardiac Valve Surgery and included 88 members. It mainly discussed: aortic valve surgery, mitral valve surgery, transcatheter techniques, valve replacement, valve repair, and minimally invasive surgery. Aortic valve disease emerged as the largest research hotspot, with the mature application of TAVR technology transforming the treatment landscape. Mitral valve repair techniques and minimally invasive techniques have also become increasingly important, representing significant developmental directions in modern valve surgery. The primary cited article in this cluster was “Guidelines for reporting mortality and morbidity after cardiac valve interventions,”^26^ cited 35 times. This research is particularly important for evaluating the outcomes, advantages, and potential risks of different surgical methods, making it an essential reference for cardiac valve surgery.

The third largest cluster (#2) was named Coronary Artery Surgery and included 82 members. It mainly discussed: graft research, coronary artery bypass grafting surgery, radial artery research, internal thoracic artery research, and cardiopulmonary bypass technology. The primary article in this cluster was “Two internal thoracic artery grafts are better than one,”^36^ cited 24 times.

### Citation Bursts Analysis

Figure 4, *C*, shows the 25 references with the strongest citation bursts in this field. During the past 5 years, Mack MJ^35^ and Popma JJ,^37^ achieved the strongest citation bursts. Both studies evaluated TAVR versus surgical treatment in low-risk patients with severe aortic stenosis. This indicates that TAVR-related articles have recently attracted intense interest from researchers, and these references are still in the burst phase.

## Key Terms

### Co-Occurrence and Clustering Analysis

**Figure 5:**
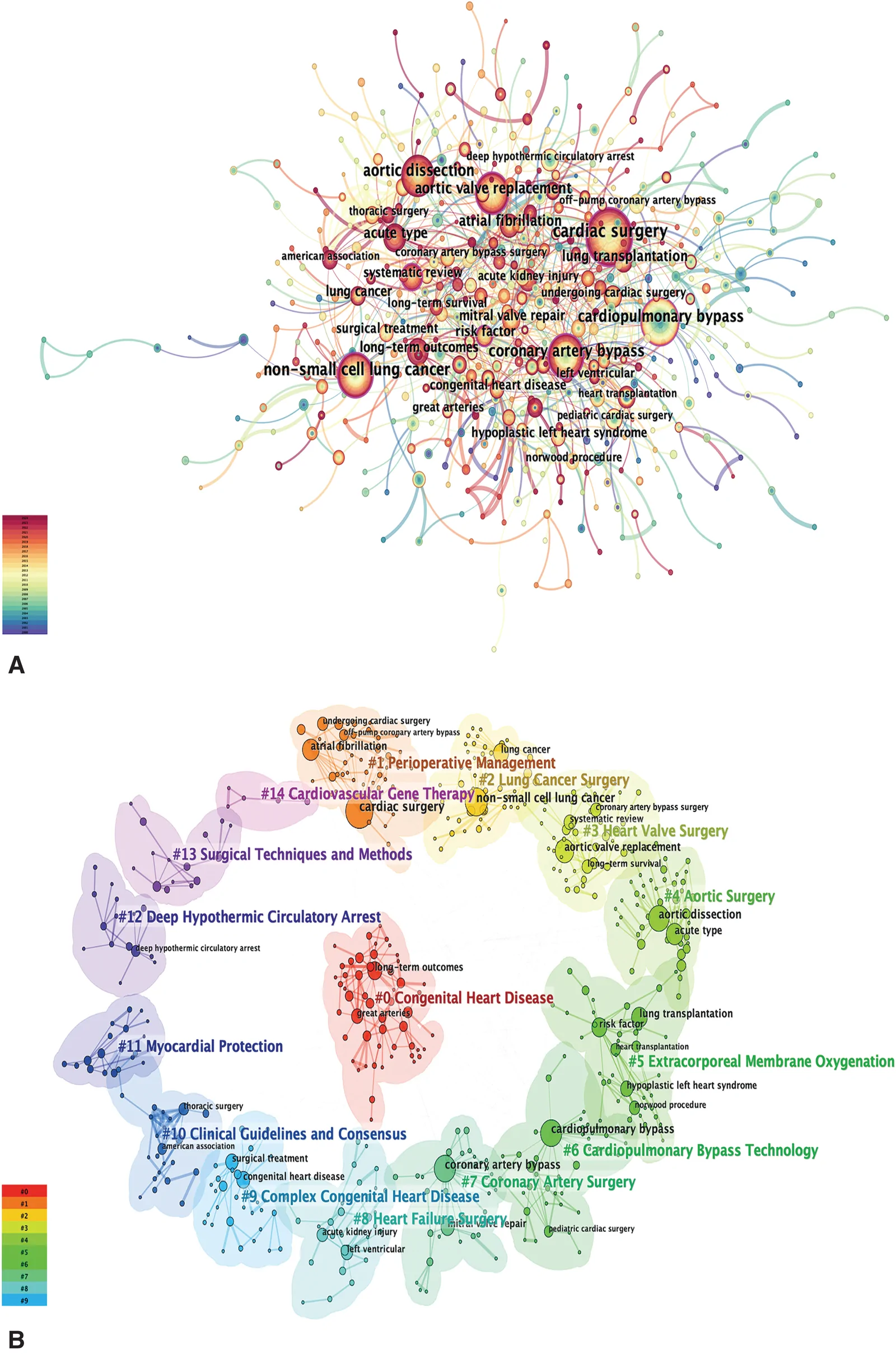

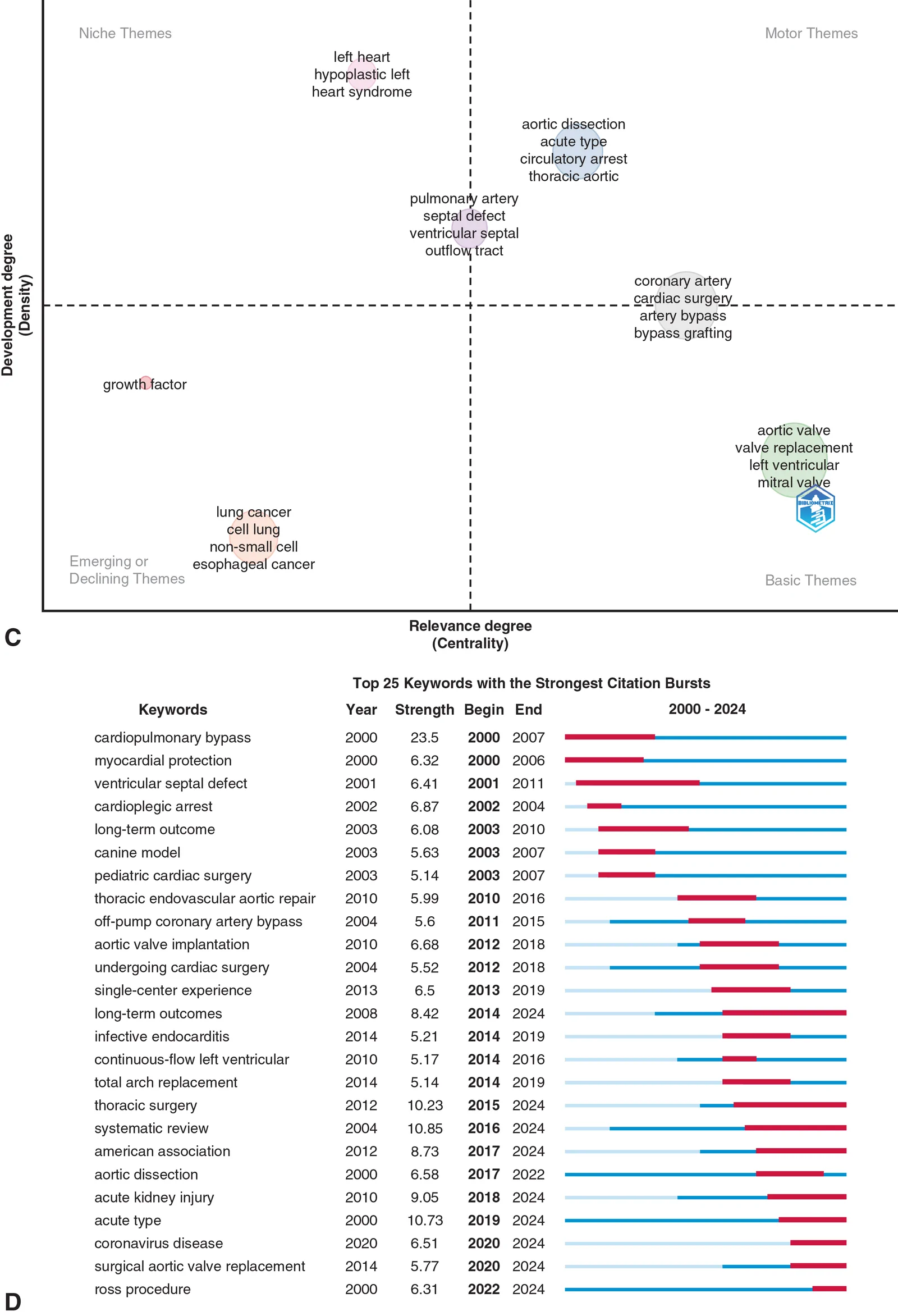
A, Visualization of the key terms in *Journal of Thoracic and Cardiovascular Surgery* (*JTCVS*). B, Clustering visualization of the key terms in JTCVS. C, Thematic map in JTCVS. D, Top-25 key terms with the strongest citation bursts.

Co-occurrence analysis examines frequently appearing key words and terms in literature to reveal their relationships. It aims to identify research themes, map connections between studies, understand past research trends, and predict future research directions. A term co-occurrence analysis was conducted on 9190 articles from 2000 to 2024, extracting 740 high-frequency terms and 1060 connections. Figure 5, *A*, presents the term co-occurrence network. Table E2 presents the top-30 key terms. Among all key terms, *cardiac surgery* exhibited the highest frequency with 300 occurrences and betweenness centrality of 0.14. This was followed by *non–small cell lung cancer*, *coronary artery bypass*, *cardiopulmonary bypass*, *aortic dissection*, *aortic valve replacement*, and *atrial fibrillation* with frequencies of 192, 188, 181, 170, 143, and 112, respectively. Except for *atrial fibrillation* with betweenness centrality of 0.08, these terms achieved betweenness centrality of 0.1 or higher, demonstrating their nodal importance. *Acute kidney injury*, first appearing in 2010, also demonstrated high frequency, reflecting increasing attention to this perioperative complication.

In CiteSpace, closely related topics are grouped into the same cluster. Figure 5, *B*, shows the key terms clustering analysis, which generated 15 clusters. The analysis encompasses major technical areas and research directions in cardiothoracic surgery. Analysis of these clusters reveals that the top-3 clusters contain 53, 51, and 51 key terms respectively, representing the 3 major research domains in cardiothoracic surgery.

### Research Trends and Key Terms Burst Analysis

The thematic map distinguishes 4 theme categories based on centrality-density relationships.^38^ We employed thematic term analysis to explore changes in JTCVS research trends shown in Figure 5, *C*. The basic themes identified in JTCVS publications were aortic valve, valve replacement, left ventricular, and mitral valve. The motor themes were aortic dissection, acute type, circulatory arrest, and thoracic aortic, whereas coronary artery fell between motor and basic themes. The niche theme was hypoplastic left heart syndrome. Figure 5, *D*, displays the top-25 key terms with the strongest bursts, revealing frequently appearing key terms within specific timeframes and thereby illustrating dynamic changes and advances in the research field. The earliest term to exhibit a burst was *cardiopulmonary bypass* (strength = 23.5; 2000-2007). The most recent burst terms included *acute kidney injury*, *acute type*, *coronavirus disease*, *surgical aortic valve replacement*, and *Ross procedure*.

## Discussion

### Journal Academic Influence

The analysis of JTCVS's publication history reveals 3 distinct developmental stages. The expansion phase (2008-2014) coincided with the rapid development of transcatheter and minimally invasive techniques in the field. This period was also the most prolific for high-influence research; Table 2 shows that 35% of the top-20 most-cited articles were published between 2009 and 2011. This indicates that the surge in publication volume was accompanied by the release of highly influential studies. In the subsequent stabilization phase (2015-2024), whereas the publication volume plateaued, the impact factor continued to rise until its peak in 2021. This divergence reflects a strategic editorial shift toward high-impact original research by redirecting clinically valuable but less-cited formats (eg, case reports) to secondary journals, solidifying JTCVS's leadership. Relative performance confirms this: whereas the impact factors of JTCVS and primary peers (*Annals of Thoracic Surgery* and *European Journal of Cardio-Thoracic Surgery*) universally peaked in 2021, JTCVS consistently maintained the highest impact factor (Figure 1).

### Global Research Landscape and Collaboration Patterns

The analysis reveals clear stratification in global cardiothoracic surgery research. At the country level, the United States maintains an overwhelming lead in both productivity and citation influence, contributing nearly half of the total output among the top-20 countries. It is followed by Japan and Canada, which also demonstrate significant contributions. In terms of collaboration, the United States shows the highest intensity, whereas Germany and Canada maintain strong international partnerships relative to their publication volumes. Notably, despite their substantial research output, major Asian contributors such as Japan and China appear relatively peripheral in the collaboration network, suggesting significant potential for broader cross-border collaboration. At the institutional level, North American institutions dominate the rankings. Harvard University and its affiliated institutions, alongside the University of Toronto, occupy the leading positions. This prominence of established academic medical centers reflects the resource-intensive nature of cardiothoracic surgery research, which favors large, well-funded ecosystems. At the author level, key figures such as Blackstone EH and Sundt TM demonstrate exceptional influence. The top-3 authors collectively account for a disproportionately high share of total citations, highlighting a significant concentration of academic influence within a small cohort of thought leaders. Furthermore, the analysis underscores a significant disparity in the global academic landscape. The dominance of North American and European institutions contrasts sharply with the underrepresentation of other regions.

### Research Evolution and Emerging Trends

The analysis of JTCVS's 20 most-cited articles (2000-2024) showed a significant concentration of high-influence publications (35%) during 2009-2011, coinciding with major advances in minimally invasive techniques. Geographically, the United States dominated with 70% of influential studies, followed by Germany and Canada at 10%. The research showed a clear clinical focus, with clinical outcome comparisons of surgical approaches (10 articles) representing the predominant theme. This clinical orientation was further reinforced by the minimal presence of basic science articles (only 1 among the top 20) and the strong influence of foundational clinical tools, particularly the RACHS-1 risk adjustment model and bicuspid aortic valve classification system. Methodological innovations and surgical technique comparisons consistently generate the most enduring scholarly influence in this surgical specialty. This trend reflects the field's applied nature but also points to opportunities for enhanced translational research integration.

The co-citation analysis of references revealed the foundational knowledge structure of cardiothoracic surgery research. Three seminal works demonstrated particularly high betweenness centrality, indicating their pivotal role as conceptual bridges across the field.^26^, ^27^, ^28^ These studies—comparing clinical outcomes of various cardiac valve treatment approaches, percutaneous coronary intervention versus coronary artery bypass grafting outcomes, and on-pump versus off-pump techniques—established critical evidence for surgical decision making that continues to influence contemporary practice. The reference clustering analysis identified 20 distinct research domains, with 3 dominant clusters characterizing the field's knowledge structure: Aortic Surgery, focusing on TAVR advancements and surgical techniques for complex aortic pathologies; Cardiac Valve Surgery, highlighting the paradigm shift toward transcatheter and minimally invasive approaches; and Coronary Artery Surgery, reinforcing evidence-based graft selection strategies. Citation burst analysis revealed TAVR as the most dynamic research frontier, with Mack MJ (2019) and Popma JJ (2019) showing the strongest recent citation bursts. These studies on TAVR in low-risk populations illustrate how technological innovations both expand clinical applications and propel research advancement in this surgically specialized field. Furthermore, the prominent citation of TAVR studies partly reflects substantial cross-disciplinary interest from major cardiology journals. Key term co-occurrence analysis has revealed the fundamental architecture of cardiothoracic surgery research. *Cardiac surgery* emerged as the most frequent keyword, first appearing in 2000 with high centrality. Other high-frequency terms, including *non–small cell lung cancer*, *coronary artery bypass*, and *cardiopulmonary bypass* collectively constitute the primary technical framework of cardiothoracic research. *Acute kidney injury* rapidly gained prominence after its relatively late emergence (2010), reflecting the field's growing emphasis on perioperative complications. Basic themes (Aortic Valve, Valve Replacement, Left Ventricular, and Mitral Valve) represent the traditional core domains that serve as critical bridges connecting various research branches. Motor themes (Aortic Dissection, Acute Type, Circulatory Arrest, and Thoracic Aortic) demonstrate well-established research systems with strong theoretical-practical integration, driving current research progress. Highly specialized areas like hypoplastic left heart syndrome have formed relatively independent research systems with tight internal connections. Recent bursts include terms *acute kidney injury*, *coronavirus disease, surgical aortic valve replacement*, and *Ross procedure,* indicating that basic topics such as traditional valve surgery still occupy an important position.

### Limitations

Several limitations should be acknowledged in this analysis. The exclusion of editorial materials, proceedings papers, and letters may overlook their important contributions to the research system. The use of title-derived terms may not fully capture the research content's complexity. Another consideration is that different bibliometric software may produce varying results due to their distinct algorithms. To ensure a comprehensive analysis, we employed 3 different bibliometric tools combined with the Citation Report directly from the Web of Science. We analyzed institutional affiliations exactly as indexed by WoSCC without manual merging. Although this ensures objectivity, it may fragment publication counts for large academic umbrellas like Harvard. Finally, bibliometric indicators, including publication counts and citation numbers, may be influenced by differences in subspecialty size and authors' career durations within the study period.

## Conclusions

This bibliometric analysis of JTCVS over 25 years (2000-2024) offers valuable insights into the evolution, current state, and future directions of cardiothoracic surgery research. The United States dominates the global research landscape, with emerging technologies such as TAVR alongside traditional valve, aortic, and coronary surgery domains representing the most dynamic frontiers. Cardiothoracic surgery has evolved into a specialized discipline with distinct yet interconnected research clusters. Future directions should focus on enhancing international collaboration and promoting research in low- and middle-income countries while integrating translational research and continuing to advance transcatheter valve technologies and minimally invasive surgeries. Simultaneously maintaining a balance between clinical advancement and fundamental research is crucial for the sustained progress of this important surgical specialty.

### Webcast

You can watch a Webcast of this AATS meeting presentation by going to: https://www.aats.org/resources/25-years-of-the-journal-of-tho-12591.

**Figure:**
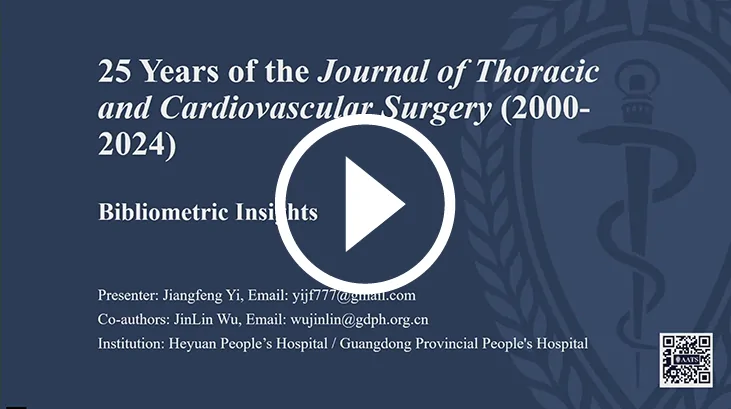

## Conflict of Interest Statement

The authors reported no conflicts of interest.

The *Journal* policy requires editors and reviewers to disclose conflicts of interest and to decline handling or reviewing manuscripts for which they may have a conflict of interest. The editors and reviewers of this article have no conflicts of interest.

## Figures and Tables

**Table E1:** The top-10 reference articles in *Journal of Thoracic and Cardiovascular Surgery* (J Thorac Cardiovasc Surg)

No.	**Paper**	DOI	Author	Journal	IF	Year	Cited times
1	2014 AHA/ACC guideline for the management of patients with valvular heart disease: A report of the American College of Cardiology/American Heart Association Task Force on Practice Guidelines^30^	10.1016/j.jtcvs.2014.05.014	Nishimura RA	J Thorac Cardiovasc Surg	4.4	2014	80
2	Transcatheter vs surgical aortic-valve replacement in high-risk patients^33^	10.1056/NEJMoa1103510	Smith CR	N Engl J Med	78.5	2011	50
3	Transcatheter aortic-valve implantation for aortic stenosis in patients who cannot undergo surgery^31^	10.1056/NEJMoa1008232	Leon MB	N Engl J Med	78.5	2010	47
4	Guidelines for reporting mortality and morbidity after cardiac valve interventions	10.1016/j.jtcvs.2007.12.002	Akins CW	J Thorac Cardiovasc Surg	4.4	2008	35
5	Comparison of shunt types in the Norwood procedure for single-ventricle lesions	10.1056/NEJMoa0912461	Ohye RG	N Engl J Med	78.5	2010	32
6	Transcatheter or Surgical Aortic-Valve Replacement in Intermediate-Risk Patients^32^	10.1056/NEJMoa1514616	Leon MB	N Engl J Med	78.5	2016	31
7	Two-year outcomes after transcatheter or surgical aortic-valve replacement^34^	10.1056/NEJMoa1200384	Kodali SK	N Engl J Med	78.5	2012	31
8	Transcatheter Aortic-Valve Replacement with a Balloon-Expandable Valve in Low-Risk Patients^35^	10.1056/NEJMoa1814052	Mack MJ	N Engl J Med	78.5	2019	30
9	Comparing apples and oranges	10.1067/mtc.2002.120329	Blackstone EH	J Thorac Cardiovasc Surg	4.4	2002	29
10	2017 ESC/EACTS Guidelines for the management of valvular heart disease^29^	10.1093/eurheartj/ehx391	Baumgartner H	Eur Heart J	35.6	2018	28

*IF*, Impact factor; *N Engl J Med*, *New England Journal of Medicine*; *Eur Heart J*, *European Heart Journal.*

**Table E2:** The top-30 key terms in *Journal of Thoracic and Cardiovascular Surgery*

Key term	Frequency	Centrality	Year	Key term	Frequency	Centrality	Year
Cardiac surgery	300	0.14	2000	hypoplastic left heart syndrome	57	0.03	2000
Non–small cell lung cancer	192	0.12	2000	systematic review	55	0.12	2004
Coronary artery bypass	188	0.15	2000	left ventricular	52	0.03	2000
Cardiopulmonary bypass	181	0.16	2000	great arteries	48	0.03	2000
Aortic dissection	170	0.06	2000	long-term survival	45	0.06	2000
Aortic valve replacement	143	0.1	2000	acute kidney injury	45	0.03	2010
Atrial fibrillation	112	0.08	2001	undergoing cardiac surgery	44	0.01	2004
Acute type	109	0.06	2000	coronary artery bypass surgery	42	0.02	2000
Lung transplantation	97	0.04	2000	Norwood procedure	41	0.01	2003
Lung cancer	88	0.05	2000	thoracic surgery	39	0.02	2012
Risk factor	85	0.1	2001	heart transplantation	33	0.03	2000
Long-term outcomes	75	0.06	2008	deep hypothermic circulatory arrest	32	0.02	2000
Mitral valve repair	71	0.05	2003	pediatric cardiac surgery	31	0.01	2003
Congenital heart disease	65	0.05	2000	American Association	30	0.03	2012
Surgical treatment	57	0.07	2000	off-pump coronary artery bypass	30	0.01	2004
